# Extensive Fever of Unknown Origin (FUO) Workup to Unmask Pheochromocytoma

**DOI:** 10.7759/cureus.15218

**Published:** 2021-05-24

**Authors:** Harpreet K Rai, Kalpana Reddy

**Affiliations:** 1 Internal Medicine, Northwell Health Long Island Jewish Forest Hills Hospital, Forest Hills, USA; 2 Endocrinology, Northwell Health Long Island Jewish Forest Hills Hospital, Forest Hills, USA

**Keywords:** fever of unknown, pheochromocytoma, adrenal, cancer, incidentaloma, metanephrines, interleukin (il)-6, adrenal gland neoplasms, hypertension, extra-adrenal paraganglioma

## Abstract

Pheochromocytoma is a tumor arising from chromaffin cells of the medulla of adrenal gland and secretes excessive amounts of catecholamines: epinephrine and norepinephrine. It can also arise from sympathetic ganglia when it is referred to as catecholamine-secreting paragangliomas or extra-adrenal pheochromocytoma. Pheochromocytoma has been referred to as “the masquerader” for its numerous atypical presentations, which makes its diagnosis medically challenging. Here, we present a case of a 66-year-old female, presenting with high-grade fever for two weeks associated with generalized body aches. She had an extensive infectious, rheumatological and hematological workup. Ultimately, she was diagnosed with pheochromocytoma. After adrenalectomy, her fever and body ache resolved.

## Introduction

Pheochromocytomas are rare neoplasms, with an estimated annual incidence of 0.8 per 100,000 person-years [1]. The annual incidence is likely to be an underestimate as autopsy studies suggest an 8% prevalence of adrenal masses and of these 4.2% are diagnosed as pheochromocytoma [2]. Pheochromocytoma accounts for 5% of adrenal incidentalomas [3]. The most common age of presentation is fourth to fifth decade with equal incidence in males and females [4].

The classic triad of pheochromocytoma consists of episodic headache, tachycardia, and sweating. It is often associated with paroxysmal hypertension. It accounts for 0.5% of cases of hypertension [5]. Among the symptomatic cases, 90% of the patients report headache and 60-70% have reported generalized sweating [6,7].

The adrenal medulla contains the enzyme phenylethanolamine-N-methyl transferase (PNMT) which converts norepinephrine to epinephrine [5,8]. Pheochromocytomas not only vary in their enzymatic composition but also in their ability to self-metabolize the catecholamines within each tumor's secretory vesicles [5]. Therefore, this could lead to great variability in the amount and ratio of catecholamines secreted by pheochromocytomas [5,9]. These differences in secretions of epinephrine, norepinephrine, and dopamine explain the heterogeneity in clinical behavior of pheochromocytomas [5]. While most of the clinical manifestations are thought to be due to excessive catecholamine release, it is postulated that these tumors also secrete a wide variety of biologically active neuropeptides, hormones, and cytokines, e.g., IL-1, IL-6, and TNF which induces various atypical clinical manifestations [10].

Diagnostic workup with either 24-hour urine fractionated metanephrines and catecholamines, or with plasma fractionated metanephrines drawn from indwelling cannula following 30 minutes of supine rest is recommended. The level of catecholamines and their metabolites in the plasma and urine provide 95% of the evidence of the disease [11]. Plasma levels of free metanephrines have a high sensitivity (99%) and specificity (89%) in diagnosing pheochromocytoma [2]. Interfering medications such as tricyclic antidepressants, nasal decongestants, etc. should be tapered off prior to testing for pheochromocytoma.

## Case presentation

A 66-year-old female with a past medical history of hypertension and chronic headaches presented with intermittent high-grade fevers for two weeks with a maximum oral temperature of 102.1 F. It was associated with generalized body aches and weakness. Her home medications were amlodipine 10 mg once a day and lisinopril 20 mg once a day. The review of systems was unremarkable. She did not have any COVID-19 exposure. Family history was unremarkable. On a physical exam, she was noted to be in mild distress due to body aches but was benign otherwise. She was febrile with a temperature of 102F, blood pressure of 156/92 mm Hg, heart rate of 110/min, and saturating well on room air. Results of blood work done on admission are shown in Table 1.

**Table 1:** Admission blood work results

Analyte	Result	Reference range
Leukocyte count	16.01 K/µL	3.80-10.50 K/µL
Hemoglobin	11.6 g/dL	11.5-15.5 g/dL
Platelet count	531 K/uL	150-400 K/uL
Sodium, serum	137 mmol/L	135-145 mmol/L
Potassium, serum	4.9 mmol/L	3.5-5.3 mmol/L
Chloride, serum	98 mmol/L	96-108 mmol/L
Total bilirubin	1.4 mg/dL	0.2-1.2 mg/dL
Alkaline phosphatase (ALP)	275 U/L	40-120 U/L
Aspartate Aminotransferase (AST)	72 U/L	10-40 U/L
Alanine Aminotransferase (ALT)	119 U/L	10-60 U/L
Total Serum Protein	9.1 g/dL	6.0-8.3 g/dL
Serum Albumin	2.2 g/dL	3.5-5.5 g/dL
Lactate	1.3 mmol/L	0.7-2.0 mmol/L

COVID-19 polymerase chain reaction (PCR) was negative. Urine analysis and chest X-ray were unremarkable. A computed tomography (CT) scan of the abdomen and chest with intravenous contrast was negative for any source of infection. It showed heterogeneously enhancing left adrenal mass measuring up to 4.3 cm in the greatest dimension. Blood cultures were sent, and she was started on piperacillin/tazobactam.

The endocrinology team was involved in the workup of adrenal incidentaloma. Her plasma renin level was 5.6 pg/ml (normal: <33.2 pg/ml) and aldosterone level was 4.1 ng/dL (normal: <23.2 ng/dL). The team decided to hold off on further workup for hypercortisolism and pheochromocytoma during hospitalization as the stress and infectious etiology can alter the results. At this point, infectious and rheumatological causes were high on differential therefore further workup for adrenal incidentaloma was deferred to the outpatient setting.

Her blood cultures were negative for any bacterial growth. QuantiFERON, hepatitis panel, and HIV were negative as well. Transthoracic echocardiogram was unremarkable. She continued to spike high-grade fever despite being on broad-spectrum antibiotics. She had an indium scan on day 6 of the hospital course, it resulted as normal. The patient was also simultaneously worked up for rheumatological causes of fever. Estimated sedimentation rate (ESR) was noted to be 101 mm/hr (normal: 0-20 mm/hr) and C-reactive protein (CRP) was 29.18 mg/L (normal: 0-10 mg/L). Given the normal Indium scan and continuous high-grade fever, antibiotics were discontinued on day 7, and the patient was started on steroids for possible rheumatological causes of fever. As she had a slight elevation in liver function test, autoimmune liver pathology was being suspected. Smooth muscle antibody was negative. Ultrasound liver showed normal appearance of liver. The patient continued to spike fever despite being on steroids. Given normal rheumatologic workup and as she continued to spike fever despite being on steroids, autoimmune pathology was unlikely at this point. Though unlikely, she was also ruled out for pulmonary embolism or deep vein thrombosis. As she had elevated total protein and low albumin, multiple myeloma was also ruled out with normal serum and urine protein electrophoresis. The patient also had a CT neck to rule out other primary malignancies with possible adrenal metastasis. The CT scan neck was unremarkable.

After infectious, rheumatological, and hematological causes were ruled out, on day 9 of the hospital course, the team decided to work her up for pheochromocytoma. Results are shown in Table 2.

**Table 2:** Lab result for pheochromocytoma workup

Analyte	Results	Reference
Plasma normetanephrine	660.7 pg/ml	0.0-191.8 pg/ml
Plasma metanephrine	<10.0 pg/mL	0.0-88.0 pg/mL
Urine normetanephrine	3174 mcg/24 hr	138-521 mcg/24 hr (normotensive), <900 mcg/24 hr (hypertensive)
Urine total metanephrine	3388 mcg/24 hr	171-616 mcg/24 hr (normotensive), <1300 mcg/24 hr (hypertensive)

**Figure 1:**
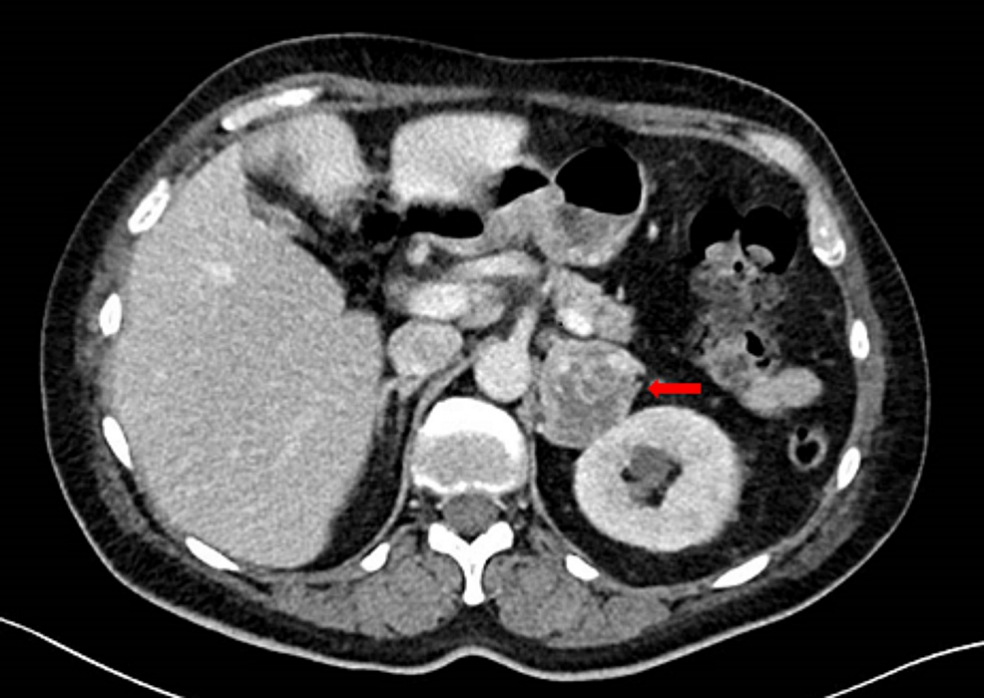
Adrenal mass noted on CT scan

An adrenal-protocol, contrast enhancement CT scan of the abdomen (Figure 1) was done. It measured approximately 4.2 cm x 3.7 cm on the axial image with 30 Hounsfield units on the pre-contrast study with a post-contrast heterogeneous pattern of enhancement. She was noted to have Interleukin 6 (IL-6) level of 126.2 pg/mL (0.0-15.5 pg/mL). The patient was diagnosed with pheochromocytoma and was started on Doxazosin 1mg at bedtime for pre-operative preparation. Two days after starting alpha-blockers, she was noted to be orthostatic positive. Volume expansion with intravenous normal saline and salt tabs was started. Metoprolol was added after four days of alpha blockade. She was discharged home with a plan for adrenalectomy in near future. She had adrenalectomy after 13 days of medical preparation and was briefly observed in the intensive care unit (ICU) post-surgery. The surgical course was uneventful. Pathology showed moderately differentiated pheochromocytoma. Post-surgery IL-6 level decreased to 29.3 pg/mL. Her fever resolved after adrenalectomy.

## Discussion

The diagnostic workup of fever of unknown origin (FUO) is one of the most vexing clinical conditions for patients and physicians. There is neither a standard approach to the diagnosis nor any published guidelines. While rheumatological diseases account for 22% of FUOs, infections and malignancies account for 16 and 7% of the cases, and 51% of cases remain undiagnosed [12].

Our patient did not have the classical clinical triad of pheochromocytoma. She had a history of hypertension, but her blood pressure was controlled with only minimal doses of amlodipine and lisinopril. During her hospital stay, she did not have any bouts of paroxysmal hypertension. The CT scan finding was significant for adrenal incidentaloma, but it was considered unlikely to be the cause of fever. As testing for pheochromocytoma and hypercortisolism in the setting of stress and infections can result in false-positive results, the team deferred the workup in the outpatient setting. After infectious, rheumatological, and hematological causes of fever were ruled out and she continued to spike high-grade fever, the team decided to work her up for pheochromocytoma.

It is hypothesized that IL-6 produced by the pheochromocytoma is causing the fevers since tumor removal led to decreased IL-6 levels and resolution of symptoms [13]. IL-6 overproduction could be either directly from the tumor or indirectly as a consequence of high levels of norepinephrine [14]. IL-6 promotes B cell differentiation, T cell activation, and induction of acute-phase reactants such as CRP. In addition to causing fever, IL-6 alters plasma protein concentration such as increasing gamma globulins, amyloid A and fibronectin or decreasing albumin. This is known as paraneoplastic syndrome. IL-6 also causes megakaryocytosis [15]. This also explains the elevated platelet count and low albumin in our patient. At the molecular level, IL-6 causes overexpression of protein kinase C-delta resulting in an excess of arachidonate derivatives such as prostaglandins, leading to systemic inflammatory syndrome [15]. Case reports have demonstrated that pheochromocytoma exhibiting pyrexia and inflammation, can be controlled by administration of nonsteroidal anti-inflammatory drugs (NSAIDs) [15]. Our patient only received acetaminophen for fever, which helped in breaking the fever. But she continued to spike a fever in the hospital course once the effect of acetaminophen weaned off. She did not receive NSAIDs during her hospitalization. It is also noted that pheochromocytoma cases with high levels of IL-6 may not show elevated blood pressure, as high levels of IL-6 cause increased nitric oxide synthesis which may lead to vasodilation [10].

Once it is diagnosed, all the patients should undergo resection after appropriate medical preparation. The management of pheochromocytoma requires a multidisciplinary approach with an internist, endocrinologist, and an experienced surgeon/anesthesiologist. Medical preparation and cardiovascular optimization are the most important aspects for surgical resection of pheochromocytoma, as the mortality can reach up to almost 50% in unprepared patients [16].

Medical optimization before surgery is aimed at controlling hypertension and volume expansion. Epinephrine acts on alpha- and beta-adrenergic receptors while norepinephrine acts on the same receptors, except beta 2 adrenergic receptors (vasodilatory action). This results in peripheral vasoconstriction by alpha receptor agonism and increased heart rate by beta 1 agonism [2]. Therefore, pre-operative medical management aims at initial alpha antagonism followed by beta antagonism. The beta-blockers should not be started first, because it will block the vasodilatory peripheral beta-adrenergic receptors with unopposed alpha-adrenergic receptor stimulation and can cause a further elevation in blood pressure. Volume contraction is associated with catecholamines and orthostatic hypotension is noted with alpha-blockers. Volume expansion along with blood pressure control is important.

Selective alpha 1 adrenergic blockers e.g., prazosin, terazosin, or doxazosin are used to control blood pressure and a diet high in sodium content (>5000 mg daily) is encouraged for volume expansion. Beta-adrenergic blockade is initiated two to three days preoperatively after adequate alpha blockage has been achieved. Metyrosine is a tyrosine hydroxylase blocker, acts to decrease the excess production of catecholamines, is rarely used when other agents are ineffective or when tumor manipulation will be marked. Calcium channel blockers (nicardipine and amlodipine) can be used to control blood pressure when alpha and beta blockage regimens fail to do so.

Surgical removal of a pheochromocytoma may not lead to a long-term cure of pheochromocytoma, even in patients with a benign tumor. In a case series of 176 patients, pheochromocytoma recurred in 29 patients (16 percent) and the recurrence was malignant in 15 of those 29 patients [17]. Mortality associated with adrenal pheochromocytoma in a large Swedish cohort study was four times higher than for controls [18].

## Conclusions

Here, we present a case of a rare diagnosis of pheochromocytoma with an unusual presentation of fever of unknown origin. Endocrine disorders are rarely a cause of FUO. Our patient was noted to have an adrenal incidentaloma on the first day of admission but the focus of the diagnostic workup was on ruling out infectious, rheumatological, and hematological causes leading to a delay in the diagnosis. It is important for clinicians to be aware of this rare presentation and include pheochromocytoma as a differential diagnosis in the workup of fever of unknown origin.
